# Perspectives of Microscopy Methods for Morphology Characterisation of Extracellular Vesicles from Human Biofluids

**DOI:** 10.3390/biomedicines9060603

**Published:** 2021-05-26

**Authors:** Mladenka Malenica, Marija Vukomanović, Mario Kurtjak, Valentina Masciotti, Simone dal Zilio, Silvio Greco, Marco Lazzarino, Vedrana Krušić, Marko Perčić, Ivana Jelovica Badovinac, Karmen Wechtersbach, Ivona Vidović, Vanja Baričević, Srećko Valić, Pero Lučin, Nika Kojc, Kristina Grabušić

**Affiliations:** 1 Department of Physiology and Immunology, Faculty of Medicine, University of Rijeka, HR-51000 Rijeka, Croatia; vedrana.krusic@medri.uniri.hr (V.K.); pero.lucin@medri.uniri.hr (P.L.); kristina.grabusic@medri.uniri.hr (K.G.); 2 Advanced Materials Department, Jožef Stefan Institute, SI-1000 Ljubljana, Slovenia; marija.vukomanovic@ijs.si (M.V.); mario.kurtjak@ijs.si (M.K.); 3 CNR-IOM Istituto Officina dei Materiali-Consiglio Nazionale delle Ricerche c/Area Scinece Park, Basovizza, I-34149 Trieste, Italy; valentinamasciotti@gmail.com (V.M.); dalzilio@iom.cnr.it (S.d.Z.); lazzarino@iom.cnr.it (M.L.); 4 A. P. E. Research srl, I-34151 Trieste, Italy; silvio.greco@aperesearch.com; 5 Faculty of Engineering, University of Rijeka, HR-51000 Rijeka, Croatia; mpercic@riteh.hr; 6 Centre for Micro- and Nanosciences and Technologies, University of Rijeka, HR-51000 Rijeka, Croatia; ijelov@phy.uniri.hr; 7 Department of Physics, University of Rijeka, HR-51000 Rijeka, Croatia; 8 Faculty of Medicine, Institute of Pathology, University of Ljubljana, SI-1000 Ljubljana, Slovenia; karmen.wechtersbach@mf.uni-lj.si (K.W.); nika.kojc@mf.uni-lj.si (N.K.); 9 Department of Biotechnology, University of Rijeka, HR-51000 Rijeka, Croatia; ivona.vidovic@student.uniri.hr (I.V.); vanja.baricevic@student.uniri.hr (V.B.); 10 Department of Medical Chemistry, Biochemistry and Clinical Chemistry, Faculty of Medicine, University of Rijeka, HR-51000 Rijeka, Croatia; svalic@medri.uniri.hr; 11 Division of Physical Chemistry, Ruđer Bošković Institute, HR-10000 Zagreb, Croatia

**Keywords:** extracellular vesicles, human biofluids, nanotechnology, atomic force microscopy, electron microscopy, morphology

## Abstract

Extracellular vesicles (EVs) are nanometric membranous structures secreted from almost every cell and present in biofluids. Because EV composition reflects the state of its parental tissue, EVs possess an enormous diagnostic/prognostic potential to reveal pathophysiological conditions. However, a prerequisite for such usage of EVs is their detailed characterisation, including visualisation which is mainly achieved by atomic force microscopy (AFM) and electron microscopy (EM). Here we summarise the EV preparation protocols for AFM and EM bringing out the main challenges in the imaging of EVs, both in their natural environment as biofluid constituents and in a saline solution after EV isolation. In addition, we discuss approaches for EV imaging and identify the potential benefits and disadvantages when different AFM and EM methods are applied, including numerous factors that influence the morphological characterisation, standardisation, or formation of artefacts. We also demonstrate the effects of some of these factors by using cerebrospinal fluid as an example of human biofluid with a simpler composition. Here presented comparison of approaches to EV imaging should help to estimate the current state in morphology research of EVs from human biofluids and to identify the most efficient pathways towards the standardisation of sample preparation and microscopy modes.

## 1. Introduction

Advanced and optimised microscopy methods are required to visualise and characterise morphology of extracellular vesicles (EVs), a heterogenous groups of nanoparticles (NPs) secreted by cells and regarded as highly promising source of diagnostic, prognostic, and therapeutic tools [1,2]. Both the choice and performance of microscopy method on one side and EV form on the other side, including whether a biofluid or a purified sample is used as an EV source, can significantly impact visualisation of EVs. Thus, we first provide description of general properties of EVs followed by an overview of EV isolation methods. Next, we describe imaging options for different electron and atomic force microscopy modes and describe the main challenges of these methods when imaging EVs present in cerebrospinal fluid (CSF) or isolated by size-exclusion chromatography (SEC). We also discuss some general aspects of sample preparation protocols, but also point to certain specificities depending on the applied microscopy mode. Moreover, sources of variation and the formation of artefacts are discussed. The critical evaluation of the published reports and terminology used for describing the shape, structure, morphology, and topography will help to estimate the current state in the morphology research of EVs from biofluids, especially from CSF, and identify the most efficient pathways for selecting, implementing, and standardising specific microscopic technologies.

## 2. Extracellular Vesicles: General Properties

EVs are nanosized phospholipid membranous structures ubiquitously found in biofluids, secreted from almost every cell, and thus reflect both physiological and pathophysiological changes of their parental cells. The lipid membrane of an EV contains proteins (e.g., tetraspanins, receptors and other molecules) and diverse luminal content with bioactive cargo that includes nucleic acids (DNA, mRNA, miRNA and lncRNA), proteins, organelles, or infectious particles [1,3]. The size distribution of EVs is between 50 and 200 nm and they possess a negative zeta potential. However, there can be variations in size, composition, and function, which in general complicate EV isolation, detection and enumeration [1,4].

EVs are classified into several subgroups based on their biogenesis or release pathway, among which exosomes have been the most widely investigated [5]. Exosomes are typically in the size of around 30–150 nm, luminal cargo is comprised of proteins, DNA, RNA, peptides and lipid derivatives surrounded by a lipid membrane. They are formed in the endocytic pathway through inward budding of endosomal membranes during their maturation into multivesicular endosomes and secreted by the fusion of multivesicular bodies with the plasma membrane. Their internalisation by recipient cells can occur through various mechanisms including endocytosis, micropinocytosis, phagocytosis, and plasma membrane fusion where its contents can influence cellular processes. They are detected on the basis of protein markers of the endosomal biogenesis pathway such as CD9, CD63, CD81 and others [6,7,8]. However, proteomic studies have demonstrated heterogeneity in this protein cargo, indicating the existence of many subclassifications of exosomes, which makes their definitive identification difficult. Exosomes released from different cells in different environments also have different membrane proteins and lipid compositions [9]. To further augment the complexity and heterogeneity of EVs, exomeres, sub-50 nm non-membranous particles have been recently discovered [1,10,11].

EVs are available as biomarker sources from biofluids such as urine, blood (serum and plasma), saliva, cerebrospinal, amniotic and other biofluids, which makes liquid biopsy an option for EV-based diagnostics [12]. Since the viscosity, fat and protein content of these fluids vary considerably, EV isolation protocol needs to be adjusted to the biofluid of interest. Additional factors that affect the amount, purity and content of EVs are diseases, use of medications, age, gender, general lifestyle, dietary habits, but also sample collection, handling and storage [1,13]. In comparison with other biofluids, CSF does not contain high concentration of NPs. NTA revealed 10^8^ of NPs per millilitre in native CSF [14], while 10^12^ per millilitre in plasma [15]. The concentration and composition of EVs in biofluids of healthy subjects may differ from the ones in patients suffering from a particular disease. Depending on the state of the disease, their cargo can contain different information about the disease [16,17]. EVs from the CSF are implicated in diverse physiological and pathophysiological processes of the brain [5]. As part of normal brain functions, they can be involved in: (i) angiogenesis, inflammation, morphogen transportation, programmed cell death, redox homeostasis and immunological functions, gene regulation, neurogenesis [18]; (ii) intercellular communication, and communication in general [19], since they can cross the blood-brain barrier (BBB) and transfer signal to other neuronal cells [2]; (iii) nerve regeneration, synaptic function, plasticity, epigenetic regulation [8,14]. Additionally, EVs can report neurological conditions and brain diseases including Huntington’s disease, Alzheimer’s disease, multiple sclerosis, Parkinson’s disease and several mental disorders, such as depression, schizophrenia, anxiety, and bipolar disorder treatment response. The activated monocytes release EVs that can influence BBB. A leaky BBB is associated with neuroinflammation, schizophrenia, bipolar disorder, and major depressive disorder [2,8,20,21]. EVs contribute or respond to pathogenic mechanisms, disease settings and progression, with cancer being the most prominent [22]. EVs serve as biomarkers to define tumours, they have pro-metastatic potential [16]. However, not all cancer cells might be using EVs to grow and metastasise, and thus not all tumours will reveal their presence by increased number of tumour-specific EVs since recent and more sophisticated methods for EV quantification, including flow cytometry and luciferase-based in vitro assays, show that certain cancer cells release lower amounts of exosomes [23,24,25]. As sources of non-invasive diagnostic biomarkers, EVs could help clinicians utilise mental health biomarkers and determine candidates for treatment strategies [26]. Furthermore, EVs can carry bioactive cargo and are thus used as therapeutic vehicles to deliver drugs or nucleic content [5,8].

## 3. Overview of EV Isolation Methods

So far, there is no gold standard for the isolation and determination of physical and biochemical characteristics of various EV populations. The isolation method is selected according to the type of the sample and the downstream analysis. Well-isolated EVs can prevent their misinterpretation and confusion with other entities of similar size (like viruses and proteins) that can also be present in complex biofluid systems. Currently, the available methods for EV isolation include ultracentrifugation (UC), ultrafiltration (UF), SEC, polymer-based precipitation and recently immunoaffinity-based precipitation [27,28].

To date, UC-based methods remain the most commonly used for EV isolation and purification. Differential UC consists of multiple centrifugation steps, which provide separation of EVs from other non-EV particles based on particle size and density. Differential UC is often followed by density gradient UC to provide additional separation of EVs from other similarly sized particles according to their bouyon density in the solution of either sucrose, iohexol, or iodixanol [27]. Although UC provides an effective way of isolating EVs, high centrifugal forces can lead to significant losses due to disruption of EV membranes, as well as a fusion of EV membranes, particle clustering and agglomeration along with the background noise [27,29,30]. Such fusion events can lead to the formation of misleading artefacts, including large heterogenous EV aggregates and multi-layered vesicles not present in the native samples [29,31]. Emelyanov et al. [32] reported EVs isolated by UC with a sizes even larger than 400 nm.

UF is another frequently used technique, both as a stand-alone or as a supplemental method for EV isolation. It is based on the separation of EVs from non-EV components in relation to their size by sieving the sample through membranes with different molecular weight cut-off (MWCO) values, using centrifugation, pressure or vacuum [27,28]. Even though UF can provide an efficient way of concentrating EVs in the sample, the applied pressure needed for the passage of particles through MWCO filter can lead to deformations and membrane ruptures. In addition, UF can cause significant losses of material as well as contamination due to the binding of EVs to the membrane and clogging of the filter pores [28,33].

Precipitation-based isolation methods have gained much popularity due to their relatively low cost and overall simplicity of the procedure. In recent years, many commercial precipitation-based EV isolation kits have become available, such as ExoQuick^TM^ (System Biosciences, Palo Alto, CA, USA), PureExo^TM^ (Qiagen, Hilden, Germany), ExoPrep (HansaBioMed, Tallinn, Estonia) and Total Exosome Isolation kit (Invitrogen, Waltham, MA, USA), which employ superhydrophillic polymers for EV precipitation [27,34]. Other precipitation protocols utilise chemicals like sodium acetate or protamine, or polymers such as polyethylene glycol to induce the precipitation of EVs. Alternatively, protein organic solvent precipitation (PROSPR) has been proposed as a method of purifying EVs by precipitation of soluble proteins present in the sample [27,35]. Although these methods provide a high EV yield, the common disadvantages include high protein contamination, resulting in overall low purity of the isolated vesicles, whilst the use of organic solvents can invoke the formation of artificially produced multilayered vesicles [36]. Moreover, retention of the polymer used for the EV isolation in the sample can interfere with further analyses and EV visualisation, e.g., Serrano-Pertierra et al. [37] reported transmission electron microscope (TEM) analysis to be unfeasible for ExoQuick EV samples due to the precipitating agent interference with the electron beam [38].

In contrast to other methods which rely on the separation of EVs from other particles based on their size, density and solubility, immunoaffinity capture offers a highly specific mode of isolation by employing antibodies against EV surface proteins [27,28]. This approach could provide isolation of EVs with high selectivity and purity, but is still not preferred due to several limiting factors. Although numerous proteins have already been associated with EVs, a protein of interest has to be expressed on the surface of EVs to be suitable for immunoaffinity capture, which narrows down the pool of potential protein candidates for this method [33]. Secondly, the presence of markers for immunoisolation in the overall EV population is often varying, which can lead to a loss of EVs that do not express the chosen protein [38]. Moreover, the detachment of EVs from the capture antibodies should also be addressed, because the elution buffers used for this process can irreversibly damage the EV membranes and cause their loss of function [28,38].

Although initially intended for the separation of peptides from amino acids, SEC has been successfully adapted for EV isolation [28]. SEC enables the separation of particles according to their hydrodynamic radius by passing the sample through a porous stationary phase. The stationary phase allows smaller particles such as proteins to enter the pores and traverse a longer path until elution, while the particles that are too big to access the pores travel faster and elute right after the void volume of the column [28,39]. The most apparent limitation regarding the SEC isolation of EVs is its inability to separate EVs from other particles of similar size, including lipoproteins and chylomicrons [39,40]. However, SEC does not seem to compromise the integrity and functionality of isolated EVs like other commonly used EV isolation methods [28,36,41,42]. SEC dilutes the sample which represents a great problem when isolating EVs from the cell culture in vitro due to the initially low NPs concentration. On the other hand, biofluids contain higher concentrations of NPs comparing to cell culture samples and dilution is not a limiting factor for them. Thus, SEC seems an ideal method for the isolation of high-quality EVs from such samples, which is necessary for investigating their biological functions.

After the isolation, a quantitative assessment (size, size distribution and concentration) is evaluated by commonly explored methods, including light scattering, tunable resistive pulse sensing (TRPS), diffraction measurement of Brownian motion (NTA), which have been reviewed elsewhere [3,43]. Detailed characterisation of size and morphology is required before any downstream investigations, e.g., functional tests, investigations of intercellular signalling pathways or clinical translation of EVs-based research [5]. To recognise and thoroughly characterise EVs in a heterogeneous sample, it is important to study individual EVs [44]. Therefore, electron microscopy (EM) and atomic force microscopy (AFM) are employed to identify and characterise individual EVs from biofluids [17,45]. Both AFM and EM are low-throughput techniques, since they allow only several particles to be observed at a time, and they give detailed information on the size, shape and deformation morphology of EVs, but in AFM also biomechanical and biophysical characteristics like adhesion, stiffness, density or elastic properties can be obtained [46,47]. The results of particle counting and size distribution should be compared with microscopy results to investigate populations that are imaged and to verify the effects of the isolation methods on morphology [43,48].

## 4. Electron Microscopy

**Figure 1:**
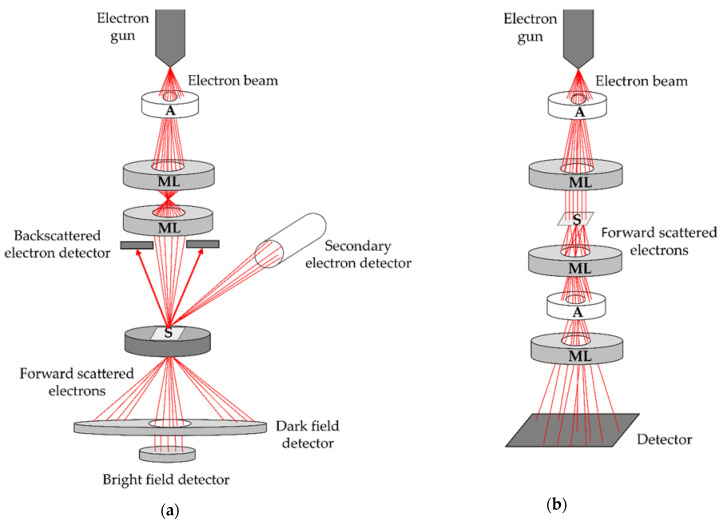
Electron microscopy. Schematic representation of scanning electron microscope (SEM and STEM—with additional detectors installed below the sample to detect the transmitted electrons; (**a**) and transmission electron microscope (TEM; (**b**). A: aperture, ML: magnetic lens, S: sample.

The size of many EVs from the biofluids is below the resolution of even the most enhanced optical microscopes. Therefore, EM is employed to assess their morphology. In general, there are two types of electron microscope: scanning electron microscope (SEM) and TEM. Both operate by irradiating the sample with a beam of electrons under a high vacuum (10^−4^–10^−5^ Pa) and rely on the interactions of these electrons with the atoms of the sample to create an image. Thus, both microscopes consist of three main components: (i) an electron gun, which produces an electron beam, (ii) magnetic lenses (ML) and apertures (A) in metal diaphragms for shaping the electron beam and guiding it to the sample (S) or a detector, and (iii) detectors, which collect the signals resulting from the interactions of the electron beam with the sample and convert them to an image or a graph (Figure 1).

The various interactions with the sample cause the electrons from the beam to change their trajectory, their energy and/or their wave properties. This phenomenon is called scattering and forward scattered electrons due to Coulomb interactions with the sample atoms are the basis of the contrast in a TEM image [49], whereas backscattered electrons (BSEs) are often used for creating high-contrast images in the SEM [50,51]. Moreover, the electron beam can excite or even eject electrons from the sample. This gives signals such as secondary electrons, X-rays or Auger electrons [49,50,51]. Electron-generated X-rays from the sample can be detected in SEM as well as TEM and enable a local analysis of chemical composition (energy-dispersive X-ray spectroscopy, EDXS) based on characteristic X-ray emission spectra of the elements.

### 4.1. Scanning Electron Microscopy

In SEM, the electrons are accelerated in the gun with voltages from a few hundred volts to 30 kV and the electron beam is focused (converged) into a probe that moves along the surface of the sample by a scanning system—scanning coils [50,51]. The detectors, which are positioned above the sample, obtain a signal from each spot during the scanning and convert the intensity of each pixel into an image (Figure 1a). For biological samples, like EVs, the secondary electron (SE) detectors are the most relevant [52]. They detect low-energy electrons that are ejected only from close to the surface of the sample and can provide valuable information about the sample (morphology and composition, texture of the surface and roughness) [50]. Hence, SEM gives information about EV surface properties, including size, shape and morphology. In some specific cases, BSE detector could also be useful, particularly with heavy-metal labelled surfaces [53]. It collects electrons that come from the greater depths under the sample surface than SEs and give information about the composition and topography [54].

### 4.2. Transmission Electron Microscopy

For TEM, the electrons are accelerated in the gun with voltages from 80 to 400 kV (or higher), the beam is almost parallel, and the sample needs to be thin enough (usually <100 nm, depending on the voltage and the sample), so that electrons can penetrate through it and reach the detector (Figure 1b). During their journey through the sample, the electrons scatter to different angles from the primary (incident) beam axis. Denser and thicker parts of the sample scatter more, likewise the parts consisting of heavier elements with a larger atomic number of the nucleus (Z) [49,55]. In this way, the so-called mass-thickness or Z-contrast is created, which is the predominant type of contrast for a biological specimen. Imaging can then be conducted in two ways. In the more common one, electrons up to a certain chosen angle are selected so the parts of the specimen, at which they were scattered more, appear darker, whereas the parts, where the electrons were scattered less, appear brighter. This type of imaging is called bright-field (BF). On the other hand, if the scattered electrons are selected instead so-called dark-field (DF) image is obtained [49]. The resolution is higher in a TEM than in a SEM. If lens imperfections (aberrations) are corrected, it can be brought down to atomic level (<1 Å). But even in a less advanced TEM, a resolution of 1 nm can regularly be achieved [49].

TEM is the most common type of electron microscopies for EV imaging [20], where it is mainly used for monitoring the quality and purity of EV-containing samples because it can better than other methods discriminate single EVs from similarly sized non-EV particles [56,57]. The specific recognition of EVs can be further enhanced by attaching immunolabels, e.g., electron opaque Au NP (with diameters ranging from 1 to 20 nm) functionalised with a specific antibody, to the antigen sites on the EVs [58]. This results in the so-called immuno-TEM imaging, which enables studying the structure functionally and observing the position of specific proteins [59]. The main disadvantage of using immunolabelling with conventional TEM is that the sample can be greatly altered during the required preparation procedure to make it immobilised and dried. On the other hand, instead of drying, biological samples can be fully solidified by freezing at very low temperatures during imaging (77 K). In that way, EVs can be observed hydrated even at high vacuum. This EM technique is called cryo-TEM, a powerful tool for assessing the morphology of EVs. It preserves the native structure of biological material and avoids alterations or modifications, commonly encountered in conventionally used EM techniques.

### 4.3. Scanning Transmission Electron Microscopy 

**Figure 2:**
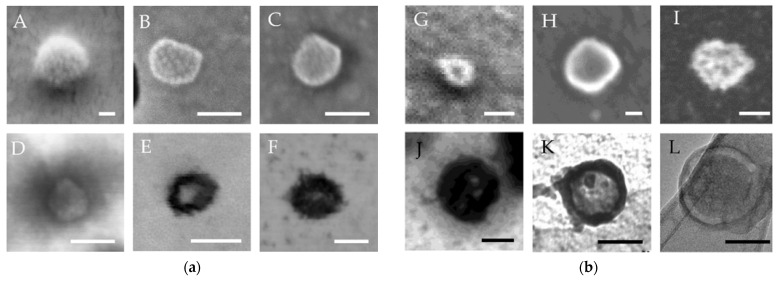
Electron microscopy of EV-like nanoparticles in native CSF (**a**) and EVs isolated by SEC (**b**), imaged in different modes. (**A**): sample fixed with PFA, on Si-wafer, air dried, tilted stage at 45°, SE; (**B**): sample fixed with GA, on polycarbonate membrane, air dried, SE; (**C**): sample fixed with GA, on polycarbonate membrane, dehydrated in ethanol gradient and critical point dried, SE; (**D**): sample fixed with GA, on carbon-formvar grid, uranyl acetate contrasted, air dried, STEM-BF; (**E**): sample fixed with GA, on carbon-formvar grid, Uranyless contrasted, air dried, STEM-BF; (**F**): sample fixed with GA, on carbon-formvar grid, UA-zero contrasted, air dried, STEM-BF; (**G**): sample fixed with PFA, on Si-wafer, air dried, tilted stage at 45°, SE; (**H**): sample fixed with GA, on polycarbonate membrane, dehydrated in ethanol gradient and critical point dried, SE; (**I**): sample fixed with PFA, on carbon-formvar grid, uranyl acetate contrasted, air dried, STEM-DF; (**J**): sample fixed with PFA, on carbon-formvar grid, uranyl acetate contrasted, air dried, STEM-BF; (**K**): sample fixed with PFA, on carbon-formvar grid, uranyl acetate contrasted, air dried, TEM-BF at 120 kV; (**L**): sample fixed with GA, on lacey carbon grid, dehydrated in ethanol gradient and critical point dried, TEM-BF at 200 kV. (**A**,**G**,**I**,**J**): Supra Zeiss 40 (Carl Zeiss AG, Oberkochen, Germany); (**B‒F**): JSM 7800F (JEOL Ltd., Tokyo, Japan); (**H**): JSM 7600F (JEOL); (**K**): JEM 1200 EXII (JEOL); (**L**): JEM 2100 (JEOL). EV: extracellular vesicle, CSF: cerebrospinal fluid, SEC: size-exclusion chromatography, PFA: paraformaldehyde, GA: glutaraldehyde, SE: scanning electron microscope—secondary electron detector, STEM-DF: scanning electron microscope—dark field detector, STEM-BF: scanning electron microscope—bright field detector, TEM-BF: transmission electron microscope—bright field detector. Scale bars represent 50 nm length in all images.

If additional detectors are installed in SEM below the sample to detect the electrons transmitted through it, BF and DF scanning transmission electron microscope (STEM) images can be created (Figure 1a). The main advantages of STEM over SEM are a higher resolution (down to <0.3 nm in the aberration-corrected instruments with field emission guns) and contrast, as well as the additional valuable information from the forward scattered electrons [60]. Hence, in case a TEM is not available, STEM can be used to ensure a more detailed analysis of EV morphology. Conventional TEM generally provides better resolution than a comparable STEM, but even in comparison with TEM (especially the ones without aberration corrections) STEM can exhibit a few beneficial properties: higher contrast, less noise in DF images and less influence by the lens aberrations [49]. Moreover, Au NP used in immunostaining can be distinguished more easily from other high-mass parts in a DF STEM image than in a TEM image [61]. Figure 2 presents examples of EV-like structures in native CSF and EVs isolated from CSF by SEC, as visualised by different EM methods under different conditions. EVs isolated by SEC from CSF were detected by protein markers of the endosomal biogenesis pathway CD9 and CD81, Supplementary Figure S1.

Several properties of EVs complicate EM investigation of their morphology and decrease the reliability of the obtained results. Electron microscopes are conventionally designed for observation of dry solid samples under high vacuum, whereas EVs are in the form of buffer suspensions after their isolation from the biofluids [62]. Moreover, EVs are non-conductive, consist of light atoms and can get easily damaged by heating. For these reasons, a special preparation procedure is necessary for observation of EVs in an electron microscope. The preparation protocol should be well selected and well optimised to keep their structural and topological characteristics as close as possible to their native form [63].

### 4.4. Sample Preparation

#### 4.4.1. Adsorption of EVs on a Substrate

The first step in preparing EVs for an EM analysis is their deposition onto a substrate. For SEM, a silicon wafer or different membrane filters are usually utilised. Silicon wafer conducts electrons and prevents charging, which enables better resolution [20]. By contrast, membrane filters are non-conductive and require sputtering with metals or carbon prior to sample observation [64].

For TEM analysis, EVs are captured on a supporting metal grid covered by a thin amorphous film/membrane that has to be electron transparent, unsusceptible to beam damage and withstand the weight of the sample [58,65]. Several types are generally encountered: (i) continuous polymer membrane, (ii) continuous carbon film and (iii) perforated (holey) polymer membrane. Continuous polymer membranes are most often made of either collodion (nitrocellulose), Formvar (polyvinyl formal) or Triafol (butyryl cellulose acetate) [65]. They are usually covered by a 5–10 nm carbon layer for reinforcement and better flow of electrons. However, this also makes them hydrophobic and impairs the capturing of the samples from the aqueous media. Therefore, electrical discharge under low pressure (glow discharge) is often used to enhance EV adhesion [66]. Perforated (holey) polymer membranes are pierced with small holes that are reinforced by sputtering with carbon. Such films must be used for high-resolution TEM (HR-TEM) microscopy and for cryo-TEM. The holes provide a cleaner background without any texture. The sample rests on the edge of the hole and is observed in the hole as if it was floating freely in the microscope [58].

#### 4.4.2. Fixation

The goal of fixation is to preserve a native form of biological sample and prevent its response to alternation of environment and conditions. There are two distinct types of fixation: (i) chemical fixation (required for SEM and conventional TEM) and (ii) cryofixation (required for cryo-TEM).

**Figure 3:**
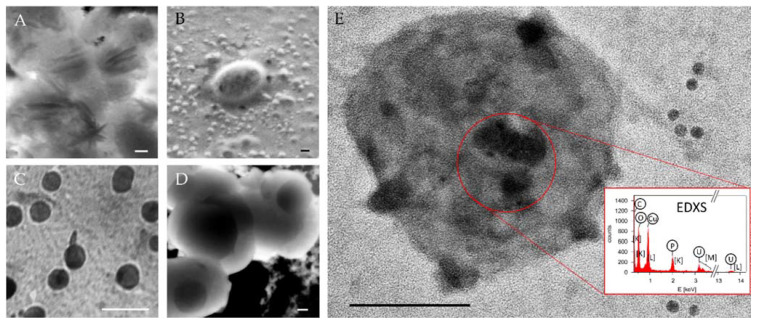
Aspects of electron microscopy in the imaging of EVs isolated by SEC from CSF. (**A**): crystal formation from interaction of phosphate buffer with the contrast solutions (uranyl acetate, OsO_4_), STEM-BF; (**B**): formation of artefacts in round shape as a result of ‘salt-bubbling’ effect, SE; (**C**): round structures with no membrane bilayer in the size of EVs, TEM-BF at 120 kV; (**D**): round structures with no membrane bilayer in the size of EVs, STEM-BF; (**E**): round structures with darker circles on the surface that could be mistaken for immunogold nanoparticles, but come from the uranyl acetate staining, as evident from the EDXS analysis, TEM-BF. (**A**,**B**,**D**): Supra Zeiss 40 (Carl Zeiss AG); (**C**): JEM 1200 EXII (JEOL); (**E**): JEM 2100 (JEOL). Scale bars represent 50 nm length in all images.

In order to protect biological samples from osmotic damage, fixatives are added into the buffer [65]. These chemicals react with the biomolecules that constitute the sample to inactivate them, cross-link them and make them less soluble, but retain the shape and structure of their connections [58,67]. Selecting the buffer during the process is critical. It should be as close as possible to the native sample environment. Nevertheless, possible interactions of the buffer with fixation and staining agents must be checked to minimise the appearance of artefacts [68]. Sometimes this cannot be easily prevented, e.g., when using SEC for EV isolation, the required mobile phase is salt buffer, which can form crystals of similar size as EVs (Figure 3A). Three different fixatives are currently mostly in use: (i) OsO_4_, (ii) formaldehyde (methylene glycol), and (iii) glutaraldehyde (GA) [69]. OsO_4_ is a strong oxidant that reacts especially with C=C double bonds of lipids, whereas aldehyde fixatives mainly react with amino groups and other nucleophilic groups (e.g., thiols) of polypeptide chains [67,70]. With its very fast and efficient cross-linking, glutaraldehyde is the best choice for the fixation of EVs. In addition, with its favourable cross-linking of membranes and introduction of a heavy metal, which obviates an additional staining step, OsO_4_ post-fixation after GA also seems a very good option, especially for the TEM analysis of EVs [70]. Nevertheless, extremely toxic OsO_4_ is much more difficult to handle than GA and over-fixation as a result of excessive crosslinking can also lead to conformational changes in membrane macromolecules, including changes in tertiary and quaternary structure of proteins [71]. The main purpose of fixation is to protect the sample against osmotic damage and additionally against the extraction of internal biomolecules, denaturation or autolysis (destruction of a cell by its own enzyme). Yet, it does not protect against the structural damage due to high surface tension during evaporation of water. Therefore, to preserve the structure, fixation has to be followed by a proper dehydration step rather than air-drying.

Immunolabelling is frequently interfering with the chemical fixation, especially with GA, which leaves many free aldehyde groups in the sample and significantly limits the accessibility to antigens [72]. This issue can be mitigated by using special antibodies that recognise glutaraldehyde linked amino acids [70], neutralising the aldehyde groups by soaking in a phosphate–glycine buffer [58], or in a blocking buffer (containing bovine serum albumin) [73], or by performing the fixation after immunolabelling.

For cryo-TEM observations, the cryofixation is the necessary prerequisite. This procedure involves very fast freezing (quenching/vitrification) of a suspension of EVs. It has to be fast enough to keep the ice crystals sufficiently small (<10 nm), so that they do not damage the structure of the sample. This can be achieved by cooling the sample to below 145 K at a rate of at least 10^3^–10^4^ K/s [58,74]. The most efficient coolant is liquid ethane [75,76], which has also been the most frequently used cryogen for cryofixation of EVs from the biofluids [29,31,32,35,36,77,78,79,80]. Cryofixation preserves the natural (realistic) structure of the sample much better than chemical fixation and it avoids or reduces the extraction or displacement of small molecules, ions and other labile or diffusible substances [58,67]. Even a combined chemical fixation never completely preserves the structure. It does not conserve saturated lipids or sugars and loosely bound ions and other very small molecules can get lost as well. Moreover, chemical fixation changes the distribution of molecules within the structure by binding together parts that were not originally bound and it adds a non-negligible number of atoms and molecules from the fixative into the structure [58].

#### 4.4.3. Dehydration

Dehydration is the final step in the biological sample preparation for conventional EM (SEM or TEM). It is a process of water removal from the sample in a way that avoids structural damage. Simple water evaporation (drying in air) is not possible, because water has a very high surface tension (around 0.07 N/m), which destroys the structure even if it is immobilised by fixation [67]. A fact that has generally been neglected even in the more recent TEM studies of EVs [81,82], in which they were simply air-dried after fixation and a characteristic “cup shape” was observed [83,84]. There are many other options besides evaporation to remove water from the sample, which can preserve its structure much better and have been used for biological sample preparation for decades. Therefore, for all EM investigations of EVs from the biofluids, the following dehydration procedures are highly recommended: (i) solvent exchange, (ii) chemical dehydration, (iii) critical point drying, or (iv) freeze-drying.

For chemically fixed samples, dehydration is most frequently done by gradually replacing the water in the sample with a solvent with lower surface tension, such as ethanol, acetone and diethyl ether [58,65,67,85]. Apart from a long solvent exchange process, water can also be chemically converted to methanol and acetone by a fast reaction with 2,2-dimethoxypropane (DMP) at pH below 6 [67,86]. Since the removal of these solvents by evaporation still does not preserve the morphology well, they should be further exchanged with some water-immiscible liquids such as tetramethylsilane (Me_4_C, TMS) or hexamethyldisilazane (Me_3_-Si-NH-Si-Me_3_, HMDS) [85,87,88,89,90,91]. An even better option is to exchange them with CO_2_ at supercritical conditions (above 31 °C and 7,3 ·10^6^ Pa), for which the surface tension vanishes [67,92]. It is important to point out that fixation is required for all these dehydration methods by solvent exchange, to protect biomolecules from coagulation, denaturation or dissolution by alcohol and other organic solvents [58]. For samples that were fixed by cryofixation and are intended for conventional (dry sample) EM, the vitreous ice can be removed by sublimation [65,74].

Immunocryo-TEM combines cryo-TEM techniques with immunogold labelling overcoming drawbacks and artefacts arising from chemical fixation and preserving EV structure and shape. Antigens can get denatured in many steps of the sample preparation for TEM so a lot of antigenicity is lost even if labelling is performed before fixation [58]. For this reason, cryo-fixation and freeze-drying, which preserve the immunoreactivity much better, are recommended in combination with immunolabelling [67]. Immunocryo-TEM has the major advantage of providing a way to identify the source cell of EVs and to immunophenotype EVs and their subpopulations in their natural hydrated environment.

#### 4.4.4. Staining

As biological samples consist of light elements (C, H, N, O, P, S) with low Z values, they create very low contrast in TEM. Therefore, microscopists traditionally try to increase the Z value of the biological samples by staining them with atoms with high Z values. There are two types of staining: negative and positive.

Negative staining denotes that there is no chemical reaction between the dye (contrast agent) and the sample, but the dye (typically a salt of heavy metal) is only adsorbed on the support film and the sample surface, so it can be easily removed by washing. It is a very fast and simple method, in which TEM grid with undried sample suspension is turned over onto a droplet of the dye solution (with the concentration of 0.5–2%) and removed after 20–30 s to prevent a chemical reaction of the contrast agent with the sample [58]. Many different contrast agents can be used: uranyl acetate (UA) (UO_2_(CH_3_COO)_2_), phosphotungstic acid (24WO_3_·2H_3_PO_4_), sodium silicotungstate (Na_4_Si(W_3_O_10_)_4_), methylamine tungstate (CH_3_NH_2_WO_3_), UA-Zero (YbCl_3_ and phosphotungstic acid), Uranyless (gadolinium and dysprosium salts), ammonium molybdate ((NH_4_)_6_Mo_7_O_24_), aurothioglucose, etc. [58]. Even though UA is becoming unattainable due to its radioactivity, it remains the preferred contrast agent for the negative staining. Due to its simplicity and usefulness for the determination of sizes and shapes of viruses, negative staining has been very popular in TEM analyses of EVs as well. Nevertheless, it can bring also many artefacts, such as size and shape changes, uneven staining, precipitation of dye crystals (Figure 3A), decreased resolution, possible mistaking grid membrane holes for sample particles etc [58,66]. It is sometimes used in immuno-TEM as well, to better see the edge of the biological sample underneath [58]. However, this can also add to the confusion, as heavy metal contrast agent precipitates can be mistaken for Au NP, especially in a BF image [61]. In this case, EDXS can be useful to help resolve the chemical nature of the dark circles (Figure 3E).

Positive staining is used when there is a chemical reaction between the contrast agent and certain parts of the biological sample, which consequently appear darker in a BF image. One such contrast agent, OsO_4_, has already been explained in detail in Section 4.4.2. A similar oxidant dye is also RuO_4_. Then some chemicals that are used for negative staining, namely phosphotungstic acid and UA, can also generate positive staining, if the sample is exposed to them for a longer time (several minutes to several hours) and/or if they are applied in higher concentrations or dissolved in other solvents than water [58]. Another very popular chemical for positive staining is lead citrate, which is produced by a reaction between lead nitrate and sodium citrate in an aqueous solution at a pH of around 12 [58]. The disadvantage of this agent is that pH has to stay above 10 during the reaction to prevent precipitation, so it has to be conducted in a sealed vessel protected from CO_2_ and containing NaOH. UA delivers good contrasting results of membranes, nucleic acids and nucleic acids containing protein complexes [93]. Moreover, it appears that lead and uranyl ions can attach to the sites already containing osmium [58]. The artefacts of this method are mainly associated with the formation of electron-dense precipitates that cover parts of the sample and can also be mistaken for biological sample particles or inorganic NP used for immunolabelling [58,61] (Figure 3E). Uranyl precipitates are usually needle-shaped, while lead precipitates are spherical [58].

#### 4.4.5. Coating

Biological samples require coating with a conductive material in order to prevent charging, i.e., electron accumulation on a non-conductive biological surface. This is usually achieved by sputtering the surface with a few nanometres (2–10 nm) [20] of metals (Au, Cr, Pt or Pd) or carbon [60]. Without coating, charging leads to image distortion (which can result in misinterpretation of the image, such as bubbles of salts appearing as spherical NP in Figure 3B) and sample damage. But even with coating, the electron beam can still damage the sample, which could be prevented by low-voltage examination [20]. Although charging can exert some damage in the TEM as well, this effect presents a problem mainly in SEM. In the TEM, the largest problem of sample damage by the electron beam is heating, so a holder cooled with liquid nitrogen (or even helium) is highly recommended [49].

Examples of different structures arising from the EV preparation for EM observation, which can result in misinterpretations during an EV visualisation, are presented in Figure 3. The use of phosphate buffer (PBS) as a mobile phase during EV separation by SEC can form crystals or spherical bubbles of similar size as EVs (Figure 3A,B). In addition, protein aggregates or lipoproteins [40] of size similar to EVs can be co-isolated in the EV fractions (Figure 3C,D). They are darker (more electron opaque) and without membranes, but especially hard to be distinguished from EVs when using conventional EM techniques with staining and possible collapse of the membrane during the sample preparation.

### 4.5. Application of EM for Characterisation of EVs from Human Biofluids

In general, SEM analysis plays an important role in determining the morphological and topographical properties of EVs, which could be correlated with the progress and the stage of the disease. Standard protocols for preparing EVs for SEM analysis are usually developed and optimised for EVs isolated in vitro and then applied to the EVs from the biofluids [64]. Due to the possible cross-interactions and structural changes after transferring EVs from their original environment to standard SEM preparation protocols, their native characteristics could be changed. Therefore, optimising the protocol for specific types and sources of EVs is a critical step towards getting precise information on their original morphology and its change during a specific disease.

The literature data on the SEM analysis of the EVs from the biofluids are very limited and only few studies have provided this kind of information (summarised in Table 1). A clear example of the effect of the isolation protocol on the EV morphological characteristics is a direct comparison of EVs isolated from the human blood using UC with the EVs subjected to the affinity column isolation protocol. The EVs, both the ones eluted from the affinity membrane and the ultracentrifuged pellet, were initially cryofixed by fast freezing in liquid nitrogen, fixed in GA (2.5 wt% in original elution buffer), dehydrated in acetone and critical point dried. SEM analysis revealed entities with sizes ranging from 50–200 nm regardless of the isolation procedure. However, in contrast to UC, for which a significant number of irregularly shaped objects were detected in the background, affinity separation was more EV specific and resulted in higher purity. Moreover, the EVs isolated using the affinity column were more regular, with rounder shape and less fused than those obtained by the centrifugation protocol [94].

In the case of cancer-sourced EVs, a study included nine patients with castration-resistance metastatic prostate cancer and twelve healthy volunteers. Cells and EVs were isolated from their peripheral blood following a centrifugation and immunomagnetic separation protocol. Then they were fixed in formaldehyde (4 wt%), dehydrated in the gradual increasing concentration of ethanol and air-dried after a further exchange with HMDS. Immunomagnetic separation using a ferrofluid conjugated with different antibodies has proven efficient for cell and EV separation from other similar objects in the complex blood system. Centrifugation enabled sedimentation of cells with cell-associated and larger micrometre-sized EVs while smaller, nano-sized EVs could not be identified. They were suggested to be additionally isolated from the remaining plasma. All circulating cancer cells were associated with EVs and the majority of them were detected on the cell surface [95].

EVs were also isolated from the saliva of healthy volunteer donors by a sequential centrifugation process in PBS [96]. The isolated EVs were deposited on UV-cleaned Si-wafers, sputtered with iridium and examined using a low–energy beam (1.5 kV). Both single and aggregated round bulging EVs without a central depression were detected, with filamentous intervesicular connections observable in their surroundings. These connections among EVs were assigned to well-preserved extravesicular channels [96].

As already described in the aforementioned examples, SEM mainly detected EVs as round structures [97] and rarely as cup-structure [98], which is probably due to better care in the preparation procedures (using critical point drying, gradual dehydration and exchange with HMDS, which are described in more detail in Section 4.4.3). Nevertheless, one study attributed the more frequent encountering of collapsed central parts in TEM also to the additional staining (or sometimes even embedding) steps for enhanced contrast in TEM, which are not needed for SEM observations [99].

TEM investigations have been conducted on EVs isolated from a variety of human biofluids (blood, saliva, urine and CSF). Such studies have been generally done at low magnifications, mainly to check the EV isolation procedure and to confirm the size distribution of EVs and their shape. Thus, TEM analyses clearly revealed that EVs from urine can be successfully isolated from the fibril network of the Tamm–Horsfall protein by the addition of dithiothreitol, which depolymerises the protein disulphide bonds [100], or by dialysis [81]. Sometimes even the concentration of EVs after their isolation from the biofluids was quantified by TEM, but the results were not reliable due to incomplete and non-uniform adhesion of EVs to the TEM grid [43,101]. Nevertheless, when Serrano-Pertiera et al. [37] compared three different isolation procedures (UC, Total Exosome Isolation kit and ExoQuick) for EVs from blood plasma, TEM images confirmed the higher yield of EV isolation for Invitrogen kit in comparison with UC. However, with the ExoQuick, precipitates of the kit interfered with the electron beam, which produced a blurred image, so the authors concluded that the ExoQuick kit is not suitable for TEM imaging [37]. Kumeda et al. [102] used TEM as one of the methods for monitoring the stability of the EVs upon storage at different conditions. Their study revealed that EVs from human saliva can retain their membrane integrity over a long storage period, even after going through freeze-thaw cycles [102].

Immunogold labelling was used in a few studies to distinguish the EVs from other round particles and to get some additional information about their structure [73,80,103,104,105,106,107,108,109,110]. Most commonly, antibodies against the tetraspanin transmembrane protein CD63 were used, which were employed for immunolabelling of EVs from blood [73,80,103,104,110] and saliva [107]. Other tetraspanins, such as CD9, have sometimes been targeted instead, for EVs from the blood [73,80,103] as well as urine [105]. Attachment of such immunogold NP to EVs indicates their exocytotic origin (they have the cytoplasmic side of the original membrane inward [105]) and also implies their possible role in signalling distant cells [103]. In relation to western blotting, many other EV-specific epitopes have been selected, such as the membrane-associated dipeptidyl peptidase IV in EVs from saliva [106] or flotillin-1 in EVs from CSF [109]. Harrington et al. [108] used 9 different antibodies to distinguish among the different EVs from the CSF that appeared in the TEM images. Cadmium selenide (CdSe) based quantum dots modified with polyethylene glycol and chemically linked to interleukin-13 (IL13QD) were prepared with the aim to identify the binding capacity of IL13QD to EVs from the CSF of the brain tumour patients isolated by differential centrifugation followed by UC [111]. Quite intriguing is also a recent study on EVs of seminal origin, in which quantum dots were covalently bonded to the primary amines on the surface of EVs via click chemistry [112]. This enabled their identification in TEM as well as a stable fluorescent labelling and live tracking. Rather than specific immunology, this labelling relies on the common fact that the membranes of EVs are rich in primary amines. Therefore, additional purification of EVs was required (using SEC after isolation by UC on sucrose cushion) to prevent labelling of proteins, and the purity of EVs was checked on the basis of EV-like NP (by NTA) to protein (by bicinchoninic acid assay) ratio [112]. Immuno-TEM has also been used to trace the origin of the EVs. Thus, a prostate-specific antigen was targeted to recognise the prostate origin of EVs from plasma [104] and antibodies against aquaporin-2, podocalyxin and podocin were used to indicate the renal origin of EVs from urine [105,113]. As evident in Table 1, no notable difference in the size and morphology of the EVs has been observed in TEM with regards to the type of biofluid, from which they originated. However, in neither of these conventional TEM studies were the EVs properly dehydrated before the examination, but merely air-dried instead. Fixation and UA negative staining have mostly been used, in some cases even the fixation step was left out [43,78,81,82]. This resulted mostly in the so-called cup-shaped (or sometimes punched-out ball-shaped) morphology. The same applies also to the STEM investigations, which have been very rare so far though. By contrast, samples for cryo-TEM imaging were always prepared according to the required procedure, by plunging them in liquid ethane, maintained at a very low temperature all the time and observed with a low electron dose in the TEM. In addition, the great structure preservation during the cryofixation and absence of drying (dehydration) enabled clear observation of the lipid membranes. The drawbacks of this method are waste of EVs because of the blotting procedure, the contrast is poor due to the absence of heavy metals, all the manipulations must be performed below devitrification temperature and particles larger than 500 nm cannot be studied [80]. The EVs (particularly the ones below 200 nm [80]) mainly exhibited perfectly spherical morphology, with a bright (electron-lucent) inside and an evident darker edge (the membrane). This was true regardless of the chosen biofluid (blood, CSF or semen, Table 1). They often appeared also as concentric circles with multiple membrane layers one around another and sometimes as larger vesicles containing one or more smaller ones inside. Although it has been speculated that such structures emerge during the EV isolation procedure (such as UC [31] or PROSPR [35], but not SEC [36]). Indeed, Issman et al. [31] have shown a decrease in the fraction of multi-layered structures upon switching from UC to filtering or dialysis. They also revealed how slow freezing and thawing of blood plasma can severely damage the EVs [31]. Moreover, Linares et al. [29] demonstrated irreversible aggregation of EVs due to UC. Nevertheless, multi-layered circles have been observed even indirectly in cryofixed biofluids without any EV isolation [79,114]. Sometimes the spherical EVs are additionally decorated with small (6 nm) spherical NP (most probably proteins) residing around 5 nm away from the surface of the EVs, which gives the so-called cauliflower-like morphology [77,79]. In a few studies, electron-dense nanospheres of similar size to the electron transparent circles were observed. These darker structures without visible membrane were ascribed to lipoproteins [32,79]. In fact, they represented the majority of the NP in a biofluid (only around 1% of them in plasma [79] and 10% in semen [114] were EVs). Recently, exomeres a sub-50 nm particles have been discovered, also a non-membranous structures [1,10].

Elongated oval and tubular particles could also be observed occasionally as well as odd-shaped particles or particles with membranes damaged during the isolation, purification and cryo-TEM preparation procedure [29,32,35,79,114]. Hence, there is a large variety of EV morphologies in every biofluid. 11 subcategories and 88 different types have been reported for human semen [114], whereas around 10 different types have been displayed in blood plasma [79] and about the same number for CSF [32]. With all these different structures it was logical to apply immunogold labelling in cryo-TEM studies as well [29,35]. Gallart-Palau et al. [35] relied on the membrane-associated CD9 and ALIX (adaptor protein for endosomal sorting complex required for transport) proteins as the epitopes, respectively, and 10 nm colloidal Au NP labelled secondary antibody to bind to them. They bound to the single-layered as well as multi-layered EVs from blood plasma [35]. A very interesting approach with double immunogold labelling can be seen in the work by Linares et al. [29], in which they used gold NP of two different sizes and functionalisations: 10 nm Au NP with antibody against integrin CD41 (or glycophorin-A CD235a) and 4 nm Au NP conjugated with Annexin A5. Double labelling on a single EV implies fusion between EVs of different cell origin or transfer of membrane-bound antigens between EVs [29].

Despite the possibility to directly analyse morphology and structure of EVs, EM techniques are generally considered as too localized, they take information from a small, usually statistically non-relevant size of the sample and are hardly reproducible [123]. However, in combination with other methods and strictly following optimised preparation protocols it can provide more reliable results and enable valuable information for study EVs complex role in intercellular communication and pathogenesis.

## 5. Atomic Force Microscopy

**Figure 4:**
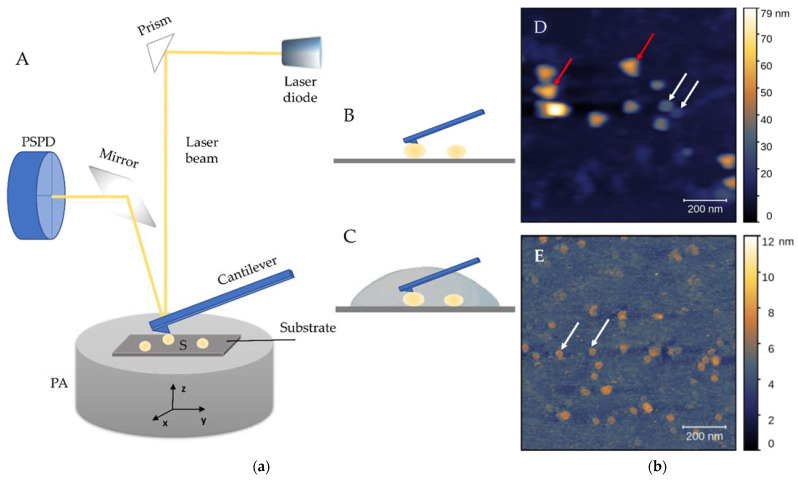
Schematic representations of atomic force microscopy (AFM, (**a**) and images of EVs isolated by the SEC (**b**). (**A**): AFM setup and detail of the tip–sample interaction in air (**B**) or liquid (**C**) environments. PSPD: position-sensitive photodiode, PA: piezoelectric actuator, S: sample. Representative images of EVs in air (**D**) and liquid (**E**). (**D)**: sample fixed with PFA, on mica, analysed using MFP-3D (Asylum Research) with OMCL/TR400PSA-HW (Olympus) probes in tapping mode, EV-like structures are pointed with white arrows and possible convolution artefacts or phosphate buffer crystals with red arrows; (**E**): sample on a glass coverslip, RCA cleaned and activated with oxygen plasma, analysed using Nanowizard II (JPK Instruments) with MLCT (Bruker) probes in tapping mode, with photothermal excitation, EV-like structures are pointed with white arrows.

AFM is a nanoscale tool for the determination of morphology, structure and composition, but also biomechanics and biophysical characteristics of nanometric structures [45,47]. Briefly, AFM uses a micrometric cantilever with a nanometre-sized tip actuated by piezoelectric crystals. Upon receiving signals of a tip-sample interaction, a position-sensitive photodiode (PSPD) converts it to a voltage and sends it to a piezoelectric actuator (PA). The latter expands and contracts proportionally to the applied voltage to manipulate the sample and the probe position across three dimensions with high precision. The PA can be coupled to a cantilever [124] or positioned under a sample holder (Figure 4(aA)). The whole system is controlled by suitable control electronics.

The laser beam is pointed on top of the cantilever and reflects onto the PSPD. When the tip passes over a topology feature, the laser beam reflects the cantilever’s motion and creates voltage signals on a PSPD. The received signal from the PSPD contains information about the cantilever’s motion which the computer tracks in x-y-z directions for the reconstruction of a topographical image [125]. The tip’s height above the surface is controlled by a feedback loop. To keep the interaction forces between the tip and the surface constant, the feedback system uses the PA to fine-tune the interaction motion in the *z*-axis direction. The position of the reflected laser beam is influenced by the exerted force and other interactions of the cantilever with the surface, but also by the frequency and amplitude of the tip’s oscillations and their combinations. Thus, besides the topography of the observed surface, its mechanical properties, e.g., stiffness, adhesion, and elasticity, can be obtained based on the measurements of interaction forces between the micro-cantilever tip and the surface of the sample at the micro- and nano-scale level.

### 5.1. Modes

**Figure 5:**
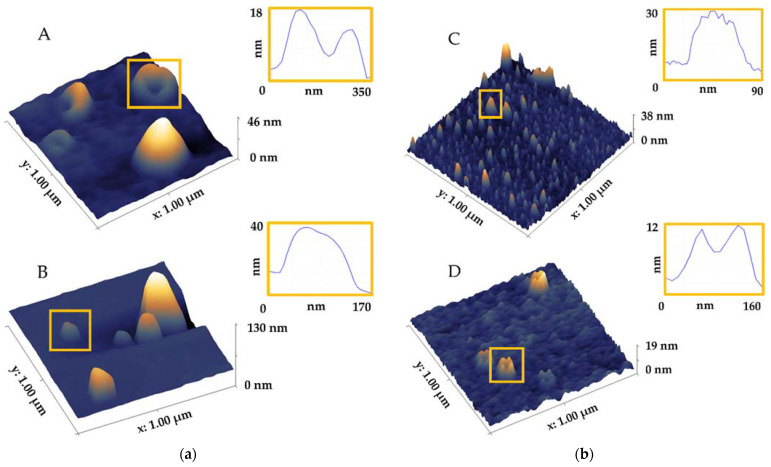
Tapping mode of AFM in the air (**a**) and liquid (**b**) environments for imaging of native CSF (**A**,**C**) and EVs isolated by SEC (**B**,**D**) with selected profiles of EV-like structures. (**A**,**B**): sample fixed with PFA, on mica, analysed using MFP-3D (Asylium Research) with OMCL/TR400PSA-HW (Olympus) probe; (**C**): sample on mica, analysed using Dimension Icon (Bruker) with ScanAsyst (Bruker) probe; (**D**): sample on a glass coverslip, RCA cleaned and activated with oxygen plasma, analysed using Nanowizard II (JPK Instruments) with MLCT (Bruker Nano) probe, with photothermal excitation.

A crucial parameter for successful imaging is the selection of the appropriate mode of operation. AFM operates in several modes: contact, tapping (or intermittent contact), non-contact and PeakForce Tapping^TM^ (Bruker, Billerica, MA, USA). The results obtained from the different modes are images or force curves [45]. When the surface of the sample is scanned, its topology is recorded from the generated signals on the PSPD (Figure 4b and Figure 5). These images can be collected in the contact mode when the tip is in continuous contact with the surface while moving in raster scan lines over a predefined scan size. The force value remains constant since the distance of the cantilever from the surface of the sample is modified for each variation of the surface height via feedback control of the PA. It provides the fastest measurements in comparison with other modes. Yet, the continuously imposed friction can damage the sample due to high lateral forces. For this reason, it is important to optimise the interaction between the tip and the sample to minimise the damage and deformation of soft, biological samples. Therefore, tapping and PeakForce Tapping modes are usually applied where more precise force control with respect to contact mode and non-contact mode is achieved.

In the tapping mode (TM) the tip oscillates across the sample surface modulated according to the sample’s height. At the lowest position of oscillation, it briefly touches the sample, thus protecting the sample from damage. The feedback loop keeps the vibration amplitude constant, and the applied force (2–10 nN) is controlled by the ratio between the amplitude setpoint and the amplitude of free oscillation. Tapping mode AFM can detect the biochemical and mechanical characteristics of the sample, its density and viscosity. The changes in the amplitude and phase of the cantilever oscillations with respect to the excitation signal can be recorded simultaneously with the topographic height.

In the PeakForce Tapping (PFT) mode the tip is used as a few pN force probe controlled by a low driving frequency and amplitude. In addition to lateral movement, the tip is forced to oscillate in the vertical direction. Unlike in TM, the piezo oscillates far below (1–2 kHz) the resonance frequency in *z*-axis direction. The maximum loading force (called peak force) perpendicular to the surface (indentation) is controlled and the computer tracking is optimised according to the peak force setpoint and the gains. The applied force is below the usually achievable values in TM [96,126,127]. It should be controlled very well for imaging of EVs, as it was observed that the shape of EV changes with the increase of the applied force [96]. While detecting the deflection of the cantilever, indentation curves or unfolding curves of a protein are collected [125]. The detected changes in the oscillation (amplitude, phase, or frequency) are used in feedback to maintain a constant interaction of the probe with the sample. The image is generated by capturing the force/distance curve on each image pixel [127,128]. The PFT mode, besides the topography, gives information about the biomechanical, biomolecular and biophysical characteristics of NP based on the force curves. The adhesion force, elastic modulus, deformation and dissipation are used for the calculation of quantitative nanomechanical information: adhesion forces, elasticity, stiffness, and deformation. EVs are exposed to different forces when attached to surfaces, during intercellular and extracellular transport, or internalisation and externalisation by cells. The measurements of biomechanical properties, the so-called mechanical fingerprint, can help elucidate these processes without the use of biochemical markers, and also give insight into adhesion-related diseases. Rigidity is one of the important properties that can distinguish different classes of EVs [47,96,129]. The central part of some EVs is softer compared to the rigid membrane and consequently a collapsed cup is formed. This can be avoided by working in very soft amplitude modulation conditions or PFT mode [130]. The membrane rigidity of EVs is generally determined by their size, lipid and protein composition, and the attractive forces on the substrate [126]. Mechanically, most EVs behave like empty liposomes with a fluid bilayer containing significant amounts of membrane-associated proteins [127].

PeakForce QNM^TM^ (Bruker**)** is an additional mode that visualises the height and the Quantitative Nano Mechanical (QNM) properties of the sample simultaneously. The first part is the PFT mode used as the feedback mode to track and image the sample surface. The QNM part uses PeakForce Tapping mode to produce force curves, from which the quantitative data on the material properties are extracted.

In non-contact mode, the cantilever oscillates at the constant distance in the close proximity above the sample, without touching its surface. As the distance is kept constant, the interaction forces between the tip and sample are in the piconewton (10–12 pN) order of magnitude (very low). By measuring either the changes in the resonant frequency or the changes in the amplitude the final image is obtained. Even though the contact forces are minimized, and the tip and the sample are preserved from the damage, the downsides of this mode are its slower scanning speed and the requirement for an ultra-high vacuum to achieve the best performance (to prevent any liquid adsorption to the tip).

There are several programs available for the postprocessing of AFM images. In particular, Skliar and Chernyshev [46] presented steps in Gwiddyion (Czech Metrology Institute, Brno, Czech Republic) for data analysis of the images of EVs. Besides the height, different parameters and characteristics, e.g., topography, morphology or root-mean-square roughness, can also be extracted.

### 5.2. Environments

Unlike EM that operates only in a vacuum and thus affects the surface morphology, AFM can operate at normal conditions and in two environments: air (Figure 4(aB),(bD)) or liquid (Figure 4(aC),(bE)) [124,126,128,130]. The air environment implies drying a sample in a gentle nitrogen stream, and it has been shown that the EVs analysed in air shrink and develop a characteristic cup shape during this evaporation process due to the central softer area collapsing with respect to the surrounding parts [84], explained in Section 4.4.3. Conversely, the liquid environment could better reflect the physiological environments in which EVs reside, under physiological conditions (i.e., in a buffer solution) and without the need for coating or any other modifications of the sample [128,131]. This better preserves the spherical shape of the EVs, but it is more complicated for the optimisation of the laser’s position and noise reduction. Hence, the imaging in the air is useful when checking the presence of vesicles and the quality of the sample. However, imaging in liquid is the optimal choice since it preserves EVs properties and reflects their natural state [125,128]. In Figure 5, different round and cup-shaped NPs in a native cerebrospinal fluid are imaged by the AFM in the air (A) and liquid (C) environments and the corresponding images of EVs after their isolation by SEC are displayed in the air (B) and liquid (D) environments. It is challenging to determine the exclusive influence of the environments on the shape of the NPs, it is rather a complex mixture of factors like different forces, the substrate applied for attaching the sample and the sample preparation protocols.

### 5.3. Cantilevers and Drives

Generally, each imaging mode will require different cantilevers depending on the measurement mode and whether the imaging is performed in liquid or in air (Figure 4(aB),(aC)). Usually, the laser power can be tuned to accommodate various drive requirements for operation in air or liquid. The cantilevers are normally mechanically driven by feeding an AC signal to a piezo transducer, but there exists a possibility for a photothermal excitation (Figure 4(bE) and Figure 5(bD)). This setting enables, in addition to the usual deflection laser beam that focuses onto the cantilever, a second blue laser used for the excitation of the cantilever to trigger its vibration. This is particularly useful for AFM imaging in a liquid. The response of the cantilever using the PA is mechanically driven by the resonances of the AFM. Thus, in the liquid environment, problems arise due to noise formation. However, with photothermal excitation, only the cantilever is excited without influencing other mechanical components in the AFM, and thus a clean response is produced, even in liquids. This method was implemented on the Cypher^TM^ family of Asylum Research AFMs (Oxford Instruments, Santa Barbara, CA, USA) and named blueDrive^TM^ photothermal excitation [132].

The resolution of the AFM depends on the size of the probe that is used to scan the surface of interest. To follow nanometric features, tips with an apex with a nanometre-sized radius of curvature are essential [125]. Also, due to the tip convolution, the particles (and other nanometric features) appear wider than they are in reality, which occurs especially when particles are of similar or smaller radii than the tip. To account for this deformation from the AFM tip in TM, deconvolution methods must be applied to calculate the actual size of the EVs, even at low forces [47]. Sebaihi et al. [133] found that for the erythrocyte-derived EVs the convolution tip effect was about 19% on the lateral measurement. The recommendation is to select tip probes of around 1 nm size for the measurements of EVs.

EVs are extremely soft, so cantilevers from silicon or silicon nitride are mainly used [127]. Low forces should be applied (the force depends on the spring constant of the cantilever), such that the EVs are minimally perturbed. Also, short interaction between the sample and the tip should be used to reduce the drift of the sample or the cup shape formation. It has been shown that an EV flattens as the force increases above 2 nN and ruptures after 5 nN [47,96]. In the case of most commonly used TM, softer cantilevers with a spring constant value lower than 0.5 Nm^−1^ are employed. For PFT cantilevers with resonant frequencies ranging between 66 and 89 kHz, spring constants ranging between 1.4 and 3.5 Nm^−1^ at a rate of 0.1–1 Hz have been used for EVs imaging [44,48,107,130,133].

AFM tip is applied also for single protein [134] and single EV investigations [96]. The single EV resolution is obtained by an antibody-coated tip and the forces perpendicular to the surface of the sample are measured as a result of the protein-antibody interactions [20,135].

### 5.4. Sample Preparation

Generally for imaging, the sample is applied onto a different flat substrate with surface roughness under 0.5 nm, which enables distinguishing the EVs from the imperfections of the surface or impurities [130]. Before attachment onto the substrate, EVs should be fixed with aldehydes (2–4% of PFA–paraformaldehyde or GA) that stabilise the nucleic acid-protein interactions and free amino groups by a cross-linking effect, which preserves the EV structure, as previously discussed in the Section 4.4.2. The materials frequently used as substrates for AFM applications are mica, glass coverslip, Si-wafer and stainless steel, or other metals. EVs are deposited on: (i) free surface, (ii) functionalised surface with a coating that binds unspecifically and (iii) antibody-coated surface for specific binding. Consequently, the EVs are adhered because of physical adsorption, electrostatic interactions, chemical bonds or hydrophobic interactions [47,126].

The mica that is freshly exfoliated to obtain a new flat layer has a residual negative charge, which makes the electrostatic interaction between the surface and the EVs sufficient to keep them attached to the surface. This applies to the imaging in the air when a washing procedure and drying under a nitrogen stream are performed. For imaging in a liquid environment, the attachment time should be prolonged, though [130].

For application on a glass coverslip, Si-wafer, stainless steel or other metals it is necessary to clean the surface of the substrate (e.g., by applying the so-called RCA standard clean, developed by Werner Kern in 1965 while working for Radio Corporation of America) [136] and create a residual charge on it, and also to enhance the binding of EVs for the substrate, e.g., by applying oxygen plasma treatment. There are also several non-specific coating solutions used for the same purpose: NiCl_2_, poly-L-lysine, GA, amine groups, peptides or aptamers [45,46,128,137,138].

EVs are negatively charged, so they bind strongly to the positively charged surfaces, such as poly-L-lysine, but not well to the negatively charged surfaces, such as unmodified mica. However, the strong adhesion of an EV to a positively charged substrate causes its structural deformation in height leading to a flattened/flat and oblate shape, so that the height/diameter ratio is much less than 1. By contrast, EVs adsorbed to mica exhibit more or less roundly shaped morphology, even though, depending on the force applied with AFM tip, it is possible that some EVs, based on their intrinsic properties (stiffness, internal structure), also exhibit some irregular morphologies, including convex, planar or concave (cup-shaped). Nevertheless, the stiffer EVs remain almost completely spherical [139].

The glass is generally selected if good optical properties are required, such as for the combined study of AFM imaging and light microscopy [130], microchip platform investigations [140], or the platform for single EV measurements combining fluorescence microscopy and AFM [138]. Gajos et al. [141] used silicon wafers with native oxidized silicon layers (SiOx) for attaching EVs generated from activated platelets and human platelet-poor plasma while Beekman et al. [44] used oxygen-treated stainless steel for multimodal analysis. There are some other examples like utilisation of 3-aminopropyltrietoxysilane (APTES), for obtaining smooth surface (named as AP-mica), also aminopropyl silatrane (APS) to yield an APS-mica surface [142] or 3-aminopropyltrimethoxysilane (APTMS) [127]. Ito et al. [126] used as-cleaned SiO_2_/Si, (APTES)-modified SiO_2_/Si, and as-cleaned TiO_2_ (as-cleaned refers to ultrasonically cleaned, placed consecutively in acetone, methanol, de-ionized water and dried in nitrogen) for attaching EVs from different cancer cell lines.

There are also different antibodies specifically used for functionalisation/coating of the surface, namely targeting membrane proteins that will capture EVs with specific epitope. The application of the antibody-coating to the surface is challenging and should include controls of the surface roughness and the presence of impurities. Hardij et al. [128] applied ethanolamine and glutaraldehyde to attach the antibody onto the substrate. Functionalisation of the surface is necessary in cases when EVs need to withstand the imaging forces (e.g., PFT), but has its pros and cons. A strong affinity of a soft vesicle for the surface can cause irreversible deformation while a weak affinity of a stiff vesicle may not be enough to retain EVs on the surface. As a result, only the specific subpopulation is imaged.

There are several antibodies applied for EVs capture. Gandham et al. [1] coated mica surface with anti-CD41 for attaching platelet-derived EVs and Sebaihi et al. [133] used anti-CD235a-modified mica for erythrocyte-derived EVs. Anti-CD142 coated mica was used to capture EVs derived from MDA-MB-231 cell line [128], anti-CD41 coated mica for EVs from platelet-poor plasma [143], anti-PAC-1-coated silicon for platelet-derived microvesicles [141]. Beekman et al. [44] used anti-EpCAM (epithelial cell adhesion molecules) to target tumour-derived EVs on stainless steel substrates previously functionalised with a monolayer of carboxydecyl phosphonic acid (CDPA) and investigated them by different methods, including SEM, AFM and Raman.

### 5.5. Aplication of AFM in Characterization of EVs from Human Biofluids

The application of AFM to investigate EVs from biofluids is still scarce. Most research has been performed on cell lines in vitro with UC as the method for isolation of EVs due to the high volume of supernatant. Recent studies point to some other methods for isolation previously discussed in the Introduction. However, discrimination of EVs from all NPs in highly complex samples like blood or plasma is challenging. EVs are outnumbered by non-EV particles (liposomes and globular proteins) that are in the same size range, which obscures and impedes the analysis of EVs. In recent years, the application of microscopy for characterisation of structure, morphology, size distribution, density, mechanical and biophysical properties of EVs, as well as their structural organisation in biofluids has advanced considerably. AFM is used for investigations of EVs in various biofluids, such as saliva, blood, plasma and serum, cerebrospinal fluid and others summarised in Table 2. Different terminology is used for describing different shapes or some morphology aspects, e.g., spherical, hemispherical, cup, ellipsoidal and flat, which creates ambiguity. It is difficult to perform a downstream analysis when these factors are not thoroughly evaluated on a single EV level [127].

The most investigated is saliva because of its availability. AFM was applied for the investigation of 2D and 3D topographic images and molecular features of EVs [107]. Pooled samples of saliva from healthy individuals were isolated by two methods: ExoQuick and UC. TM AFM imaging in air conditions revealed larger (>100 nm), heterogeneous, irregularly shaped, aggregated EVs after isolation by ExoQuick, in contrast to the homogeneous, single, round-shaped EVs that were isolated by UC. The structure and biomechanical properties were investigated for UC isolated saliva exosomes from healthy individuals at the single vesicle level [96]. TM height images in air conditions with forces below 1 nN revealed round 50–70 nm EVs. The amplitude images with applied forces around 2 nN showed EVs with similar morphology, an average diameter around 100 nm and an indent in the centre pointing to mechanical deformation by the tip. The phase images with 2 nN forces also revealed a 3D trilobed structure and substructures in the centre of the EVs with different contrast (possible from different constituents, namely lipids, proteins, nucleic acids). If high forces were applied, structural deformation and disintegration followed. The importance of the implementation of immuno-based detection methods lies in distinguishing EVs from other structures like globular proteins. It involves functionalisation of the flat surface that EVs are attached to or tip functionalisation. Sharma et al. [96] detected a single-molecule of transmembrane protein CD63 on the surface of saliva EVs imaged under PBS in TM via targeted force spectroscopy with an antibody-coated tip and antibody-labelled gold beads. This principle enables the detection of specific membrane markers for specific diseases (e.g., oral cancer) on the membrane of EVs from biofluids after mass spectrometry detection of the target protein in subpopulations of EVs. Topographic images have been applied to compare the UC-isolated exosomes from the saliva of healthy individuals with the saliva of oral cancer patients [144]. Using TM AFM in the air, the normal exosomes exhibited circular, homogeneous, bulging structure and diameter of 40–80 nm, with a distinct phase contrast between the less dense vesicle periphery and the more dense core region. On the other hand, cancer exosomes were bigger with broader distribution of 20–400 nm and manifested irregular morphologies, aggregation and clustering. Also, the larger EVs appeared hollow without the dense core region seen in the normal EVs. Furthermore, cancer exosomes indicated a possible increased surface CD63 density [144].

Urinary EVs have been found to own great potential applications in disease diagnosis, therapy and disease molecular mechanism. The urine is rich in Tamm-Horsfall protein (around 92 kDa) and other biological components. Yang et al. [81] isolated EVs by dialysing urine in 300 kDa dialysis tubes in PBS solution, and then the dialysis suspension was concentrated by using 100 kDa ultracentrifuge tubes. Samples were analysed by AFM in TM on freshly cleaved mica in air conditions and showed a round structure with no aggregation or disruption.

The erythrocyte-derived EVs, isolated by UC were imaged by TM AFM on anti-CD235a-modified mica [133]. Glycophorin A (CD235a), uniquely expressed on the erythrocyte membrane was chemically attached to mica. EVs examined under buffer in liquid and in air conditions showed similar morphology in both media, spheroidal shape, around 30 nm high and 90 nm wide. Rikkert et al. [145] investigated blood-derived EVs on a poly-L-lysine coverslip by AFM in PBS environment with PFT mode using minimal imaging force. Their topography and mechanical properties were obtained from the force-indentation curves. The particles were 25 nm high with a spherical shape. The authors pointed out that AFM imaging alone is not enough for distinguishing between EVs and lipoproteins. Therefore, an isolation protocol combining gradient- and size-based approaches is necessary to ensure the presence of only EVs [145]. Blood samples of 96 patients were investigated for the monitoring of fingerprint for CNS tumours (glioblastoma multiforme, benign meningioma and single brain metastasis originating from non-small-cell lung cancer) carried by small EVs [146]. EVs were isolated by UC and the presence of small EVs was confirmed by AFM in TM, in PBS solution. Their size range spanned from 50 to 140 nm and they appeared as various structures. The force spectroscopy measurements of EVs are still scarcely studied. Bairamukov et al. [139] found a correlation between the biomechanical properties of the EVs, their size, structure, and function. They used PeakForce QNM in air and liquid for measurements of exosomes and exomeres isolated by UC from blood plasma. This AFM mode, as mentioned earlier, acquires high-resolution (HR) AFM images with force spectroscopy measurements at the same time. The measurements of the biomechanical properties revealed a soft internal cavity that was referred to as a disk-like shape, a stiffer membrane for exosome in the liquid and near-spherical shape in the air. By contrast, exomeres had similar heights in the air and the liquid environments [139]. EVs are considered promising biomarkers for thrombotic risk. AFM imaging of EVs from platelet-free plasma on tissue factor (TF) coated mica revealed only a few vesicles, with a size range of 60–100 nm [128]. Biomechanical investigations are important for the detection of a difference between normal and cancerous EVs. Vorselen et al. [127] compared EVs from healthy individuals with the ones from the patients with hereditary spherocytosis (HS) by measuring their biomechanical properties in PFT mode, in PBS. Blood-derived EVs, which were isolated by UC and adhered to poly-L-lysine on APTMS coated glass slides, appeared as spherical structures with a mean radius of 71 nm. Furthermore, HS patient-derived EVs were significantly softened in comparison with the healthy donor-derived EVs, and their protein composition was altered.

The first-ever 3D images of NP in native CSF are presented in Figure 5(aA), on mica in the air (A) and in the liquid (C), showing two distinct structures: round and cup shape. These structures can also be visible in Figure 5(aB), representing SEC-isolated EVs on mica in the air (C) with possible convolution artefacts or PBS crystals (used as mobile phase during EV separation by SEC) and on a glass coverslip in the liquid, RCA cleaned and activated with oxygen plasma (D). Similar features are visible for the EVs isolated by SEC in Figure 4(bD),(bE) too. For exploring the impact of different parameters on the shape and morphology of EVs, additional effort should be invested in assessing the influence of the single factors, e.g., isolation method, or settings in the AFM imaging, prior to downstream analysis.

## 6. Conclusions

AFM and EM methods are powerful tools to characterise EV morphology. However, both the EV and microscopy-associated aspects can significantly affect the final imaging results and thus require detailed and dedicated optimisation. On one hand there is the milieu containing the EVs to be visualised. The milieu can be either a biofluid or a buffer after EV isolation. Each type of EV-containing milieu can have specific acidity, salt composition and concentration, all of which can significantly influence downstream procedure of sample preparation for microscopy techniques. On the other hand, AFM and EM sample preparation protocols and methods are equally possible in myriad versions and are not standardised, thus they render the microscopic characterisation of EVs variable and unreliable. Several factors cause this variability and different terminology is used for describing the variation in the shape, morphology, structure and topography of EVs. Special attention should be focused on the steps in the sample preparation protocols and on controlling the formation of artefacts. Along with the standard application of EM and AFM for revealing the presence of EV-like structures and impurities, a single-EV approach based on immuno-detection should be introduced to reduce the level of uncertainty. The sources of variation should be evaluated before any downstream analyses so that misinterpretations during the investigations of the biological functions of EVs can be prevented.

## Figures and Tables

**Table 1:** Morphological and structural characteristics of the EVs from the human biofluids as obtained by electron microscopy.

Method	Biofluid	Shape	Structure/morpHology/Topography	Size (nm)	References
SEM	blood	regular vs. Irregular spherical	aggregated, rough surface, adhered to cell surface, rough surface, non-fused vs. fused	/	[94,95]
	saliva	round—irregular	individual and aggregated, rough surface	/	[96]
STEM	blood	donut-shaped cup-shaped	/	100 (average)	[80,115]
TEM	blood	cup-shaped round oval punched-out ball	cytoplasmic side inward	30–240	[37,43,80,103,104,110,116,117,118]
	saliva	round	individual vesicles some > 200 nm and deformed aggregate formation	30–250	[102,106,107]
	urine	punched-out soccer ball round cup-shaped deformed	/	20–250	[81,82,100,101,105,113,119]
	CSF	cup-shaped spherical round	/	20 to >200 20–150	[14,43,108,109,111,120,121,122]
	semen	cap and circular	membrane structures	80–200	[112]
Cryo-TEM	blood	round, elongated filled tubular, some odd-shaped oval spherical and tubular	apparent lipid membrane, actin filaments visible, granulated, smooth, bilayered, multilayered, with smaller spherical particles at the surface, EV aggregates	20–500	[29,31,35,36,78,79,84]
	semen	round egg-shaped oval elongated (sausage-shaped) tubular	trilamellar membrane, 5 nm cauliflower-like protrusions 4 nm away from the 5 nm thick membrane, most double or triple, a few single vesicles, vesicle sacs containing 6 or more EVs, pleomorphic membrane structures, coated membranes, double membrane bilayers, some EVs more electron dense	25–500	[77,114]
	CSF	round (single, double) slightly elongated (multilayer)	single, double, multi-layered, clear presence of lipid bilayer/membrane	26–435	[32,78]

**Table 2:** Morphological and structural characteristics of the EVs from the human biofluids as obtained by atomic force microscopy.

Method	Biofluid	Shape	Structure/Morphology/Topography	Size (nm)	References
AFM air	saliva	ring-like irregular vs. round circular	with central indentation trilobed membrane individual vs. aggregated heterogeneous vs. homogeneous aggregated vs. single bulging agglomerates less dense periphery/more dense core larger vesicles without dense core clusters	50–70 around 100 >100 40–80 20–400	[96,107,144]
	urine	round	individual vesicles, no aggregation	/	[81]
	blood	spheroidal near-spherical cup-shape	increased stiffness irreversible deformation	~30 high/~90 wide 23.7 high/71.3 lateral 3.16 high/31.2 lateral 60–100	[128,133,139]
AFM liquid	saliva	ring-like	with central indentation trilobed membrane	50–70around 100	[96]
	blood	spheroidal spherical cup-shape disc-like	various structures soft inner cavity stiffer membrane softer vs. stiffer	~30 high/~90 wide ~25 high 50–140 6.26 high/70.55 lateral 4.16 high/16.3 lateral	[127,133,139,145,146]

## Data Availability

The data presented in this study are available on request from the corresponding author.

## Supplementary Materials

The following are available online at https://www.mdpi.com/article/10.3390/biomedicines9060603/s1, Figure S1: Expression of CD9 and CD81 markers in samples of EVs isolated by size-exclusion chromatography from human cerebrospinal fluid (SEC 1–4). U-87, positive control, human brain (glioblastoma astrocytoma); SH-SY5Y, positive control, human neuroblastoma.
