# Supplementary figures and images for: Perspectives of Microscopy Methods for Morphology Characterisation of Extracellular Vesicles from Human Biofluids
Source: Biomedicines. 2021 May 26;9(6):603. doi: 10.3390/biomedicines9060603 (PMC8228884; doi:10.3390/biomedicines9060603)

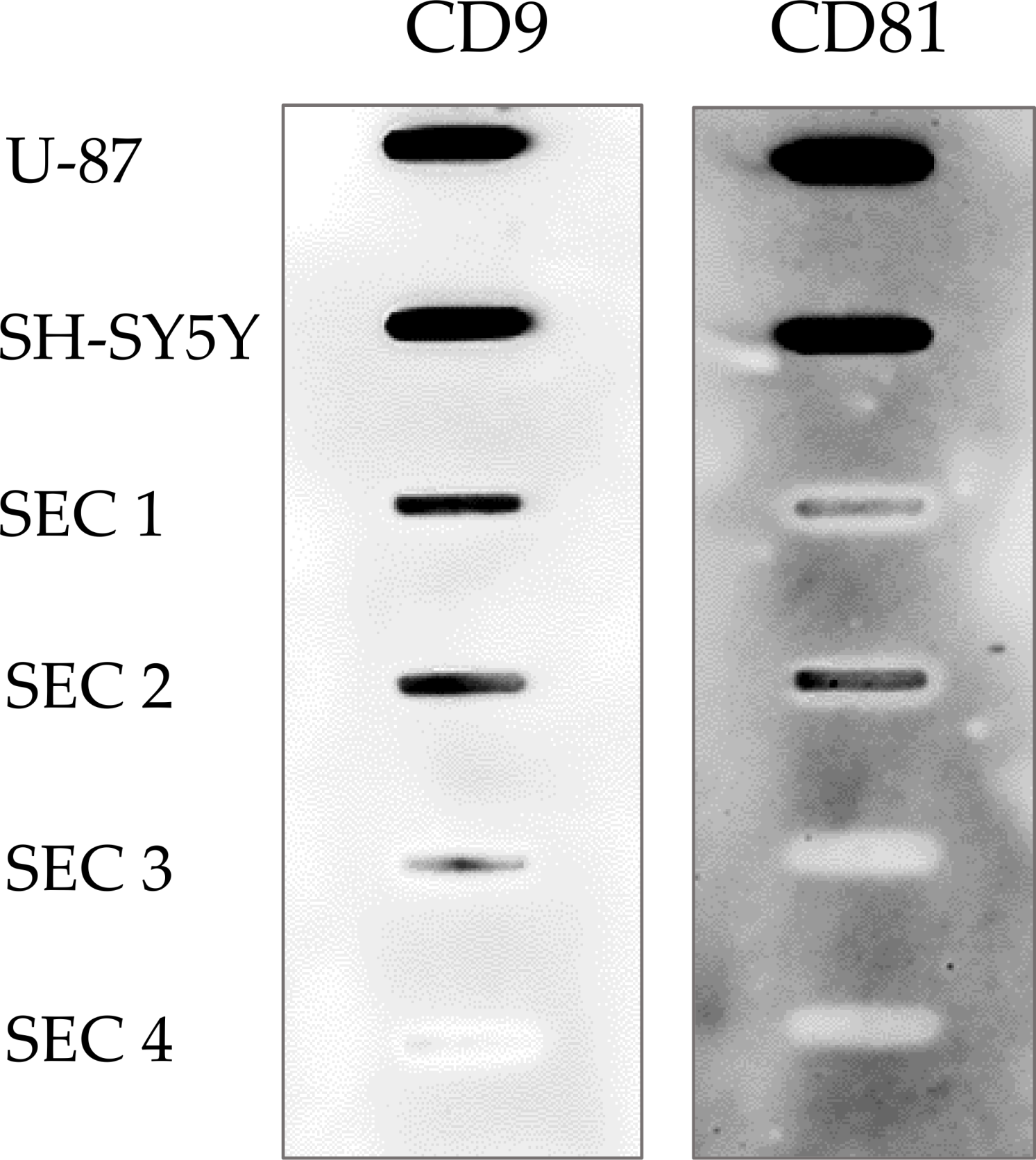

Supplement: Supplementary file 1 [file biomedicines-09-00603-s001.zip › biomedicines-1223404-supplementary.tiff]
